# Clinical Effects and Differences in Neural Function Connectivity Revealed by MRI in Subacute Hemispheric and Brainstem Infarction Patients With Dysphagia After Swallowing Therapy

**DOI:** 10.3389/fnins.2018.00488

**Published:** 2018-07-20

**Authors:** Yu-Chi Huang, Tun-Wei Hsu, Chau-Peng Leong, Han-Chin Hsieh, Wei-Che Lin

**Affiliations:** ^1^Department of Physical Medicine and Rehabilitation, Kaohsiung Chang Gung Memorial Hospital, Chang Gung University College of Medicine, Kaohsiung, Taiwan; ^2^Department of Diagnostic Radiology, Taipei Veterans General Hospital, Taipei, Taiwan; ^3^Department of Diagnostic Radiology, Kaohsiung Chang Gung Memorial Hospital, Chang Gung University College of Medicine, Kaohsiung, Taiwan

**Keywords:** dysphagia, stroke, swallowing therapy, videofluoroscopy, magnetic resonance imaging

## Abstract

**Background:** Early detection and intervention for post-stroke dysphagia could reduce the incidence of pulmonary complications and mortality. The aims of this study were to investigate the benefits of swallowing therapy in swallowing function and brain neuro-plasticity and to explore the relationship between swallowing function recovery and neuroplasticity after swallowing therapy in cerebral and brainstem stroke patients with dysphagia.

**Methods:** We collected 17 subacute stroke patients with dysphagia (11 cerebral stroke patients with a median age of 76 years and 6 brainstem stroke patients with a median age of 70 years). Each patient received swallowing therapies during hospitalization. For each patient, functional oral intake scale (FOIS), functional dysphagia scale (FDS) and 8-point penetration-aspiration scale (PAS) in videofluoroscopy swallowing study (VFSS), and brain functional magnetic resonance imaging (fMRI) were evaluated before and after treatment.

**Results:** FOIS (*p* = 0.003 in hemispheric group and *p* = 0.039 in brainstem group) and FDS (*p* = 0.006 in hemispheric group and *p* = 0.028 in brainstem group) were both significantly improved after treatment in hemispheric and brainstem stroke patients. In hemispheric stroke patients, changes in FOIS were related to changes of functional brain connectivity in the ventral default mode network (vDMN) of the precuneus in brain functional MRI (fMRI). In brainstem stroke patients, changes in FOIS were related to changes of functional brain connectivity in the left sensorimotor network (LSMN) of the left postcentral region characterized by brain fMRI.

**Conclusion:** Both hemispheric and brainstem stroke patients with different swallowing difficulties showed improvements after swallowing training. For these two dysphagic stroke groups with corresponding etiologies, swallowing therapy could contribute to different functional neuroplasticity.

## Introduction

Dysphagia is a common disorder after stroke (Paciaroni et al., 2004). Approximately 25~45% of patients show difficulties in swallowing after acute stroke while dysphagia is associated with a high risk of aspiration pneumonia, malnutrition, and mortality after acute stroke (Barer, 1989; Johnson et al., 1993; Martino et al., 2005; Walter et al., 2007; Falsetti et al., 2009). Previous studies have reported that 15% of stroke patients depend on tube feeding in order to maintain their nutritional requirements 6 months after stroke (Croghan et al., 1994; Dennis et al., 2005; Smithard et al., 2007). Consequently, the early detection and management of post-stroke dysphagia could reduce the incidence of subsequent co-morbidities and help to reduce the length of duration of hospitalization (Dziewas et al., 2004; Brotherton and Judd, 2007; Maeshima et al., 2014). Therefore, early swallowing therapy for dysphagia is very important in preventing further pulmonary complications and mortality after stroke (Doggett et al., 2001; Foley et al., 2008).

Brainstem or hemispheric lesions after stroke can lead to motor or sensory deficits in swallowing function. Brainstem stroke may also impair the sensory input or motor function of the mouth, tongue, cheek, pharynx, larynx, vocal fold, or cricopharyngeal muscles during the pharyngeal phase of swallowing (Horner et al., 1991; Moon et al., 2012). Hemispheric stroke may also impede the motor control and coordination of bolus preparation, mastication, or pharyngeal peristalsis during the oral and pharyngeal phases during swallowing. Therefore, different types of stroke may cause swallowing impairments in different ways in stroke patients with dysphagia (Robbins et al., 1993; Moon et al., 2012; Kim et al., 2014).

Several swallowing therapies have been utilized in the management of post-stroke dysphagia, including thermal stimulation, postural compensation, food consistency modifications, oropharyngeal exercises, swallowing maneuver and electrical stimulation (Carnaby et al., 2006). Recently, some investigators have used neuromuscular electrical stimulation for dysphagia patients after stroke and found this mode of treatment to be beneficial in the treatment of dysphagia (Lee et al., 2014; Poorjavad et al., 2014; Byeon and Koh, 2016). In earlier studies, some researchers reported that these therapeutic techniques might provide positive effects on neuroplasticity for stroke patients with dysphagia. Voluntary swallowing is regulated by complex sensorimotor cortical and subcortical network while the swallowing reflex is controlled by swallowing centers in the brainstem (Soros et al., 2009). However, the actual mechanism underlying swallowing function in the brain has yet to be elucidated. In stroke patients, damage to both of the associated hemispheres or brainstem result in swallowing disorders (Hamdy et al., 1996, 1997, 1998a,b; Li et al., 2009, 2014).

Previous studies have used clinical observations, EEG, magnetoencephalography (MEG) or transcranial magnetic stimulation (TMS) to explore the cerebral areas associated with swallowing dysfunction (Robbins et al., 1993; Hamdy et al., 1997; Luan et al., 2013; Jestrovic et al., 2016). However, over recent years, functional magnetic resonance imaging (fMRI) has become a more popular tool with which to evaluate cerebral cortical function during volitional and reflexive swallowing in humans (Hamdy et al., 1999; Mosier K. et al., 1999; Mosier K. M. et al., 1999; Kern et al., 2001; Martin et al., 2001; Malandraki et al., 2011; Dehaghani et al., 2016). Li et al. used fMRI to investigate cerebral cortical activation during swallowing with tasks in acute dysphagic stroke patients involving of unilateral hemisphere (Li et al., 2009). On the basis of these results, they suggested that fMRI is a useful method to investigate the spatial localization of changes in the neuroactivity of the bilateral hemispheres during swallowing tasks which may be related to the functional recovery of dysphagia (Li et al., 2009). The strongest activations were noted in the sensorimotor cortices, insula and cingulated gyrus of the intact hemisphere. Furthermore, they reported decreased connectivity in bilateral swallowing associated brain network while applying rs-fMRI study to explore the alternations of functional and structural connectivity in stroke patients with dysphagia (Li et al., 2014). Another fMRI study investigated recovered swallowing in dysphagic stroke patients and also showed overall decreased fMRI-activation in the associated swallowing network, but increased activities in the contralesional primary somatosensory cortex (S1) (Mihai et al., 2016).

Diffusion Tensor Imaging (DTI) is commonly used to study normal white matter anatomy and structural connectivity. This technique might have the potential to also assess plastic changes in the white matter in response to intense therapy in stroke patients recovering from a motor deficit. However, some of the DTI-derived absolute measures have shown pre-vs. post-therapy changes that were in the order of variability seen in normal subjects. Furthermore, significant remodeling of the ipsilesional and contralesional corticospinal tract was observed in chronic stroke patients undergoing an intense therapy program (Betzler et al., 2009). Consequently, in order to assess the sensitivity of this technique to detect possible structural changes as a function of therapy, it is necessary to carry out an assessment of the observed variance in DTI-derived measurements (Betzler et al., 2009).

To the best of our knowledge, there are limited published literature pertaining to the relationship between the changes in swallowing function and functional connectivity maps of fMRI, and in hemispheric and brainstem stroke patients following swallowing therapies. The aims of the present study were to investigate the benefits of swallowing therapy for swallowing function and brain neuro-plasticity and to explore the relationship between swallowing function and fMRI findings following swallowing therapy in hemispheric and brainstem stroke patients with dysphagia.

## Materials and methods

### Participants

Between January 2011 and July 2013, we collected 31 acute stroke patients with dysphagia who met the inclusion criteria from the inpatient rehabilitation unit at one medical center. (clinical trial No.: NCT03048916) Stroke was diagnosed by the attending neurologist according to the patient's neurological insults and findings from brain computed tomography (CT) or magnetic resonance imaging (MRI) findings. The inclusion criteria for the participants were recent hemispheric or brainstem stroke (with a duration of less than 3 months since stroke), dysphagia reported by a physician who had assessed choking or coughing during swallowing, or patients were failed to complete the 100 ml water test during bedside swallowing assessment (Chen et al., 2016). We used functional oral intake scale (FOIS) to present patient's swallowing performance, which may reflex the severity of swallowing; patients were included in the study if their FOIS was equal to or less than 4. The exclusion criteria in the study were as follows: impaired communication ability due to aphasia; cognition impairment; a history of other neurological deficits leading to dysphagia; use of an electrically sensitive biomedical device (such as a cardiac pacemaker or a metal clip in the brain). Only seventeen stroke patients with dysphagia underwent detailed clinical assessments, videofluoroscopic swallowing studies (VFSS), and fMRI examinations before and after swallowing therapy. The study protocol was reviewed and approved by the Institutional Review Broad in our hospital. Informed written consent was obtained from each participant.

### Clinical assessments and swallowing therapy

For each patient, the following clinical characteristics were recorded during admission into the rehabilitation unit: age; gender; swallowing therapy type; duration since stroke onset; the National Institute of Health Stroke Scale (NIHHS); affected hemisphere and lesion location. Finally, we recruited 11 patients with hemispheric stroke into the hemispheric group and 6 patients with brainstem stroke into the brainstem group; all of these patients underwent the entire evaluation and treatment process.

During hospitalization, these subacute stroke patients with dysphagia received traditional swallowing therapy or a combined therapy including both traditional swallowing therapies and neuromuscular electrical stimulation (NMES) therapy. Every patient was treated 3 times per week (60 min per session). Total ten treatment sessions with traditional or combined therapy were performed for each patient. The traditional swallowing therapy was performed by one experienced speech-language therapist, including oropharyngeal exercises, thermal stimulation, food consistency modifications, compensatory techniques, and swallowing maneuver based on VFSS findings and clinical presentations. NMES therapy was administered by one licensed physiatrist who used the VitalStim device (Chattanooga Group, Hixson, TN, USA) with a dual channel and two bipolar electrodes for each channel (700 μs pulse width, 80 Hz, and 0-25 mA wave-amplitude). The two sets of electrodes were placed on the patient's anterior neck area (Freed et al., 2001). Wave amplitude setting was dependent upon the patient's level of tolerance. The physiatrist gradually increased the amplitude until the patient felt a tingling sensation on the anterior neck and a muscle contraction. The current intensity was determined and maintained during one NMES treatment session based on each patient's tolerance level. The patients all underwent traditional swallowing training and NMES by the same physiatrist while receiving the combined therapies.

### Clinical swallowing function and videofluoroscopy evaluations

The functional oral intake scale (FOIS) for dysphagia was evaluated before and after intervention by a speech-language therapist who was blinded to all study procedures. The FOIS (Crary et al., 2005) is widely used for clinically assessing oral intake in stroke patients. Seven swallowing functional levels were defined according to their oral intake conditions ranging from nothing by mouth (level 1) to total oral diet with no restriction (level 7). The VFSS was performed by another speech-language therapist who was also blinded to the study interventions. The 8-point penetration-aspiration scale (PAS) (Rosenbek et al., 1996) and functional dysphagia scale (FDS) (Han et al., 2001) were assessed according to VFSS findings. The 8-point PAS indicated the severity of aspiration while swallowing. Aspiration was defined as any materials entering the larynx below the vocal folds. The 8-point PAS scale ranged from a score of 1 (normal swallowing without material entering the airway) to a score of 8 (severe airway compromise with material entering the airway and passing below the vocal folds). The FDS includes 11 items relating to oral and pharyngeal function and was investigated during the VFSS according to lip closure, bolus formation, residual matter in the oral cavity, oral transit time, triggering of pharyngeal swallowing, laryngeal elevation and epiglottis closure, nasal penetration, triggering, residue in valleculae and pyriform sinus, pharyngeal coating, and pharyngeal transit time. For each patient, the achievable score is 100 points depended upon the results of the VFSS. A higher FDS score indicated a worse swallowing condition in the VFSS. Another experienced and blinded speech-language therapist interpreted and scored the 8-point PAS and FDS before and after interventions.

### Acquisition of MRI data

Functional imaging data were acquired using a 3.0 T GE Signa MRI scanner (Milwaukee, WI, USA). Resting-state images from 300 contiguous echo planar imaging whole brain functional scans were acquired (TR: 2 s; TE: 30 ms; FOV: 240 mm; flip angle: 80°; matrix size: 64 × 64; thickness: 4 mm). During the resting experiment, the scanner room was darkened and the participants were instructed to relax, with their eyes closed, without falling asleep. A three-dimensional (3D) high-resolution T_1_-weighted anatomical image was also acquired using an inversion recovery fast spoiled gradient-recalled echo pulse sequence (TR: 9.5 ms; TE: 3.9 ms; TI: 450 ms; flip angle: 20°; field of view: 256 mm; matrix size: 512 × 512).

### Resting-state functional MRI (rs-fMRI) preprocessing

Rs-fMRI data were preprocessed using Statistical Parametric Mapping (SPM8, Wellcome Department of Cognitive Neurology, London, UK; http://www.fil.ion.ucl.ac.uk/spm/) and Data Processing Assistant for rs-fMRI (DPARSF) and in-house software for Matlab (The MathWorks Inc. Natick MA, USA).

In the first step, the first 5 volumes were discarded to reach a steady-state magnetization and allow the participants to adapt to the scanning noise. We excluded any data involving a head motion of more than 2.0 mm maximum displacement in any of the x, y, or z directions, or 2.0° of any angular motion throughout the course of the scan. Data were also visually inspected for movement-related artifacts. The standard Montreal Neurological Institute template provided by SPM was further used for normalization with re-sampling to 3 mm cubic voxels and a Gaussian kernel of 6 mm (full width at half maximum) for spatial smoothing. The waveform of each voxel was finally used to remove linear trends of the time courses and for temporal band-pass filtering (0.01–0.08 Hz) to reduce high-frequency physiological noise.

### Independent component analysis of rs-fMRI networks

To investigate rs-fMRI networks, we performed independent component analyses (ICA) using group ICA for fMRI toolbox (GIFT; http://mialab.mrn.org/software/gift). This toolbox supports a group ICA approach, which first concatenates the individual data across time, followed by computation of the subject-specific components and time courses. Data dimensionality (the number of components) was estimated using the minimum description length criteria tool in GIFT (Calhoun et al., 2001), which suggested that 30 was the optimal number of independent components (ICs). The dimensions of the functional data were then reduced using principal component analysis and the ICs were then estimated using the Infomax algorithm (Bell and Sejnowski, 1995). Stable estimation was achieved by rerunning the ICA analyses 20 times using the ICASSO (Himberg et al., 2004; Chenji et al., 2016) toolbox implemented in GIFT. Twenty-four ICs with an ICASSO stability index of <0.9 were then estimated in our patient sample and the spatial maps of the components were converted into *z*-value maps (Stevens et al., 2009). Further analysis of ICs were selected based on the largest spatial correlation (Calhoun et al., 2001) with specific rs-fMRI network templates reported in previous studies (Shirer et al., 2012), which correspond to known anatomical and functional segmentation. Group statistical maps of subject IC patterns representing rs-fMRI networks were entered into one- and two-sample random effects analyses in SPM8.

### Statistical analysis

SPSS package (version 17.0, Chicago, IL, USA) was used to run statistical analysis for group differences in demography, clinical characteristics, and VFSS findings. The Mann-Whitney test was used to analyse data relating to age, the duration since stroke, and NIHSS, and while Fisher's exact test was used to analyse gender, swallowing therapy types, and the affected hemisphere between the brain and brainstem groups. Within-group and between-group comparisons of FDS, 8-point PAS, and FOIS were performed with the Wilcoxon signed-rank test and Mann-Whitney test. The significance threshold of group difference was *p* < 0.05, FDR-corrected for multiple comparisons. Functional connectivity (FC) between-group two sample t tests were masked with a within-group mask threshold at *p* < 0.05, and corrected for multiple comparisons using a combination of an uncorrected height threshold of *p* < 0.05 with a minimum cluster size. Cluster size was determined over 1000 Monte Carlo simulations using the AlphaSim program distributed with the REST software tool (http://resting-fmri.sourceforge.net/). Anatomical labeling was defined by the Anatomical Automatic Labeling atlas (AAL) (Tzourio-Mazoyer et al., 2002). In voxel based statistical analysis, some significant clusters between groups were found. To evaluate their interactions with the clinical symptoms, the connectivity value was extracted from those significant clusters for further correlation analysis. The correlation between changes in clinical parameters and the alteration of functional connectivity was investigated using Spearman‘s rank correlation.

## Results

### Demographic and clinical characteristics

We initially collected 31 acute stroke patients with dysphagia but excluded 14 patients who showed poor cooperation during the VFSS/ fMRI study or swallowing training. Twenty-three out of 31 stroke patients received full-course swallowing training during hospital stay. Five of those 23 participants were not suitable to receive fMRI assessments and one of 23 patients could not completely take all 3 kinds of food in VFSS. In this study, we did not find any exacerbations of the medical conditions including aspiration or pneumonia during swallowing training and no harm related to all procedures for each participant. Consequently, a total of 17 patients completed all of the procedures and interventions required by this study.

Of the 17 patients included in the study, there were 11 patients with hemispheric stroke assigned to the hemispheric group and 6 patients with brainstem stroke assigned to the brainstem group. The demographic and clinical characteristics of these participants are shown in Table 1. In the hemispheric group (2 women and 9 men; median age: 76 years), 7 patients received combined therapy and 4 patients received traditional swallowing therapy. In the brainstem group (1 woman and 5 men; median age: 70 years), 4 patients received combined therapy and 2 patients received traditional swallowing therapy. There were no significant differences in terms of age, swallowing therapy type, duration since stroke onset, NIHSS, and affected hemisphere. Within-group comparison (Table 2) showed significant differences in terms of thin-liquid FDS (*p* = 0.012), total FDS scores (*p* = 0.006), FOIS (*p* = 0.003) in the hemispheric group after treatment, and significant differences in soft diet FDS (*p* = 0.043), thin-liquid FDS (*p* = 0.046), total FDS score (*p* = 0.028), PAS (*p* = 0.041), and FOIS (*p* = 0.039) in the brainstem group after treatment. Between-group comparison showed significant differences in thick-liquid FDS (*p* = 0.049) and FOIS (*p* = 0.039) after treatment. There was also a significant difference between the 2 groups of patients after intervention in terms of their relative changes on the 8-point PAS (*p* = 0.028) (Table 3).

**Table 1:** Patients characteristics.

	**Patient**	**Age**	**Gender**	**Swallowing therapy**	**Duration since stroke onset (days)**	**NIHSS**	**Aff. Hem**.	**Lesion location**
Hemisphere Group	1	81	F	Traditional	33	13	L	Frontal, temporal, and parietal lobes
	2	67	F	Traditional	40	8	R	Basal ganglia, frontal, temporal, and parietal lobes
	3	64	M	Traditional	15	13	L	Thalamus, corona radiate, and occipital lobe
	4	73	M	Traditional	10	3	L	Basal ganglia and corona radiata
	5	77	M	Combined	19	11	L	Basal ganglia and corona radiata
	6	75	M	Combined	11	10	R	Corona radiata and parietal lobe
	7	70	M	Combined	35	11	L	Frontal and parietal lobes
	8	58	M	Combined	12	10	L	Basal ganglia
	9	54	M	Combined	17	5	L	Basal ganglia
	10	60	M	Combined	8	6	L	Basal ganglia
	11	57	M	Combined	8	15	R	Frontal lobe
Brainstem Group	12	64	F	Traditional	14	4	R	Lower medulla
	13	71	M	Traditional	17	12	L	Basal pons
	14	56	M	Combined	36	3	R	Pontomedullary junction
	15	76	M	Combined	11	8	L	Pons
	16	52	M	Combined	41	7	R	Pontomedullary junction, lower pons, cerebellum, and occipital lobe
	17	40	M	Combined	24	12	R	Paramedium pons

*Patients characteristics including age at fMRI study date, gender, swallowing therapy, duration after stroke onset, NIHSS, affected hemisphere, lesion location. Gender: M, male; F, female; Affective hemisphere (Aff. Hem.): L, left; R, right; NIHSS, National Institute of Health Stroke Scale*.

**Table 2:** A comparison of findings in the VFSS and clinical oral intake conditions within and between hemispheric and brainstem groups.

	**Hemispheric group (*****n*** = **11)**			**Brainstem group (*****n*** = **6)**			***p*1**	***p*2**
	**Before treatment**	**After treatment**	***p***	**Before treatment**	**After treatment**	***p***		
**FDS**								
Soft diet (median [IQR])	18.0 (14.0)	12.0 (13.0)	0.120	22.5 (22.25)	11.5 (22.25)	0.043^*^	0.228	0.613
Thick liquid (median [IQR])	21.0 (20.0)	16.0 (16.0)	0.066	25.5 (22.5)	29.0 (15.5)	0.463	0.189	0.049^*^
Thin liquid (median [IQR])	16.0 (11.0)	12.0 (5.0)	0.012^*^	23.5 (18.0)	19.5 (27.0)	0.046^*^	0.288	0.311
Total FDS score (median [IQR])	63.0 (43.0)	38.0 (30.0)	0.006^*^	69.5 (66.75)	55.0 (53.0)	0.028^*^	0.291	0.132
8-point PAS (median [IQR])	2.0 (4.0)	1.0 (1.0)	0.121	5.5 (4.75)	2.0 (4.25)	0.041^*^	0.149	0.239
FOIS (median [IQR])	3.0 (3.0)	7.0 (1.0)	0.003^*^	1.0 (0.5)	3.5 (3.25)	0.039^*^	0.070	0.039^*^

*Within-group and between-group comparisons of FDS, 8-point PAS, and FOIS were performed by the Wilcoxon signed-rank test and Mann-Whitney test. VFSS, videofluoroscopic swallow study; FDS, functional dysphagia scale; PAS, penetration-aspiration scale; FOIS, functional oral intake scale; IQR, interquartile range. p: comparison between before and after treatment in each group; p1, comparison between two groups before treatment; p2, comparison between two groups after treatment*.

* *p < 0.05*.

**Table 3:** A comparison of the changes in clinical outcome measures between the hemispheric and brainstem groups.

	**Hemispheric group (*n* = 11)**	**Brainstem group (*n* = 6)**	***p***
**FDS**			
Soft diet (median [IQR])	3.0 (24.0)	5.5 (6.25)	0.688
Thick liquid (median [IQR])	2.0 (12.0)	4.5 (16.0)	0.481
Thin liquid (median [IQR])	4.0 (14.0)	1.5 (16.5)	0.448
Total FDS score (median [IQR])	−13.0 (41.0)	−16.0 (11.75)	1.0
8-point PAS (median [IQR])	0 (2.0)	1.5 (1.75)	0.028^*^
FOIS (median [IQR])	3.0 (1.0)	2.5 (1.75)	0.104

*Between-group comparisons of the changes in FDS, 8-point PAS, and FOIS were performed by the Mann-Whitney test. FDS, functional dysphagia scale; PAS, penetration-aspiration scale; FOIS, functional oral intake scale; IQR, interquartile range*.

* *p < 0.05*.

### Components of the resting-state functional networks

**Figure 1:**
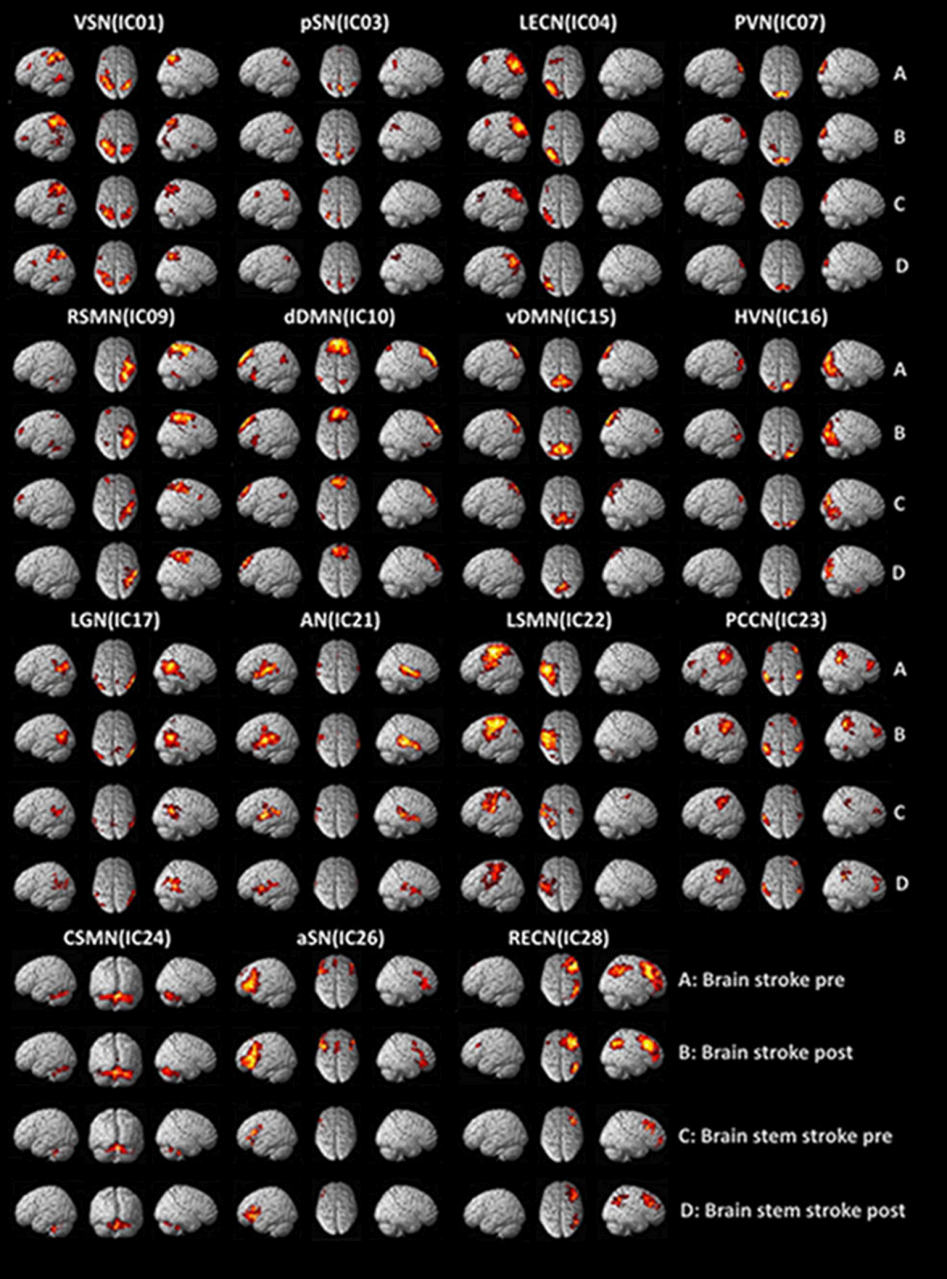
Functional MRI findings in stroke patients with dysphagia before and after swallowing training. VSN, visuospatial network; pSN, posterior salience network; LECN, left executive control network; PVN, primary visual network; RSMN, right sensorimotor network; dDMN, dorsal default mode network; vDMN, ventral default mode network; HVN, high visual network; LGN, language network; AN, auditory network; LSMN, left sensorimotor network; PCCN, precuneus network; CSMN, cerebellar sensorimotor network; aSN, anterior salience network; RECN, right executive control network.

Rs-fMRI networks were identified by using 14 templates in all ICA components for each of the 17 subjects. Correlation coefficients between the spatial templates and ICs of ICA analysis were as follows: visuospatial network, VSN (IC01), 0.292; posterior salience network, pSN (IC03), 0.474; left executive control network, LECN (IC04), 0.469; primary visual network, PVN (IC07), 0.398; right sensorimotor network, RSMN (IC09), 0.284; dorsal default mode network, dDMN (IC10), 0.471; ventral default mode network, vDMN (IC15), 0.263; high visual network, HVN (IC16), 0.330; language network, LGN (IC17), 0.330; auditory network, AN (IC21), 0.414; left sensorimotor network, LSMN (IC22), 0.264; precuneus network, PCCN (IC23), 0.368; cerebellar sensorimotor network, CSMN (IC24), 0.272; anterior salience network, aSN (IC26), 0.313; and right executive control network, RECN (IC28), 0.492. The results of the one-sample *t*-test (*P* < 0.05, FDR corrected) for patients with hemispheric and brainstem stroke were determined using SPM toolbox and are shown in Figure 1.

### Functional connectivity in rs-fMRI networks: group comparison

We compared FC between patients with hemispheric and brainstem stroke in a voxel-wise manner. Significance differences arising from two-sample *t*-tests (between the two groups) and paired *t*-tests (between the two states) of spatial map regions are presented in Table 4.

**Table 4:** Functional connectivity differences in rs-fMRI networks: a group comparison.

**Groups**	**Network or component label**	**AAL cluster labels**	**Cluster size**	**Peak T-score**	**Peak MNI coordinate**		
					***x***	***y***	***z***
**HEMISPHERIC GROUP**							
Before treatment > after treatment	vDMN (IC15)	Precuneus_L	51	3.647	0	−51	54
	SMN (IC24)	Cerebelum_Crus1_L	53	4.014	−30	−81	−24
Before treatment < after treatment	VSN (IC01)	Parietal_Sup_L	166	1.813	−21	−57	51
	dDMN (IC10)	Frontal_Mid_R	83	3.732	21	60	27
	SMN (IC24)	Cerebelum_6_R	148	3.508	21	−57	−33
**BRAINSTEM GROUP**							
Before treatment > after treatment	SMN (IC09)	Postcentral_R	55	4.030	42	−39	72
	vDMN (IC15)	Parietal_Sup_L	121	4.692	−15	−78	45
		Precuneus_L	57	6.458	−6	−57	54
	HVN (IC16)	Fusiform_R	249	11.797	27	−54	−12
	AN (IC21)	Temporal_Mid_L	80	10.589	−51	−48	12
	SMN (IC22)	Postcentral_L	53	6.033	−66	−12	9
	PCCN (IC23)	Parietal_Inf_L	69	6.853	−63	−51	36
Before treatment < after treatment	PVN (IC07)	Cerebelum_6_L	70	7.991	−9	−63	−9
		Calcarine_R	100	5.499	15	−69	15

*AAL, Automated Anatomical Labeling; MNI, Montreal Neurological Institute stereotaxic coordinate; IC, independent components; vDMN, ventral default mode network; SMN, sensorimotor network; VSN, visuospatial network; dDMN, dorsal default mode network; HVN, high visual network; AN, auditory network; PCCN, precuneus network; PVN, primary visual network*.

The hemispheric group with post-intervention exhibited significantly increased intra-network functional connectivity in the left superior parietal lobule of the VSN, the right middle frontal cortex of dDMN, and the right cerebellum of SMN.

The brainstem group with post-intervention exhibited significantly increased intra-network functional connectivity in the left cerebellum and right calcarine of the PVN. In contrast, the brainstem group with post-intervention exhibited significantly reduced intra-network functional connectivity in the right postcentral cortex of the SMN, left superior parietal lobule of the vDMN, left precuneus of the vDMN, right fusiform of the HVN, left middle temporal lobe of the AN, left postcentral cortex of the SMN and left inferior parietal of the PCCN.

### Relation between rs-fMRI networks and clinical parameters

**Figure 2:**
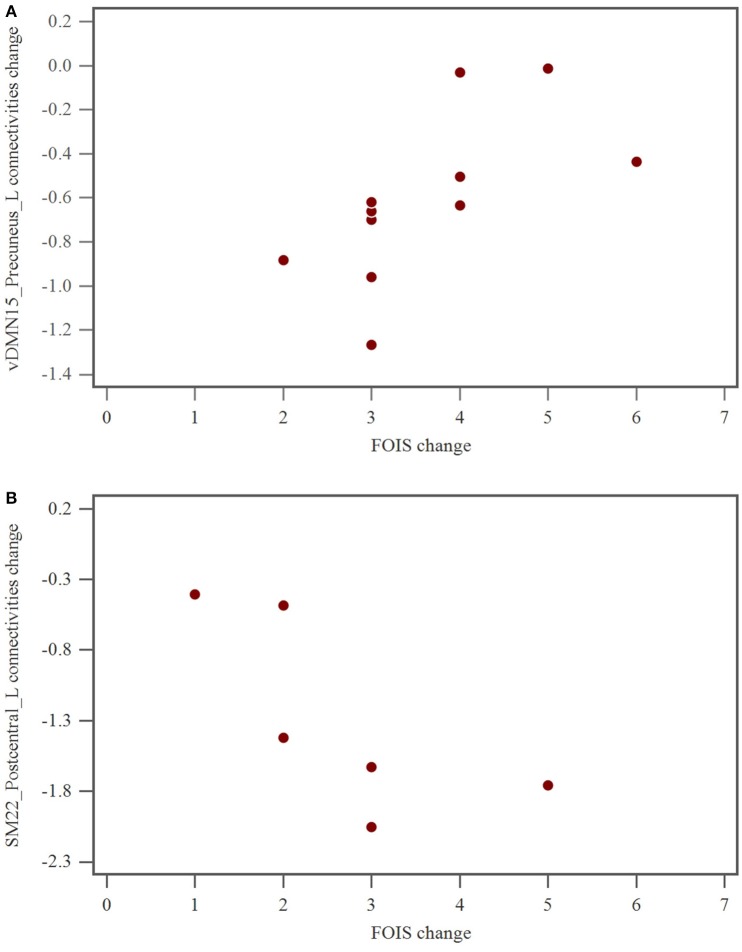
**(A)** The relationship of FOIS change and vDMN15_Precuneus_L connectivity alteration after swallowing therapy in hemispheric group. FOIS, functional oral intake scale; vDMN, ventral default mode network. **(B)** The relationship of FOIS change and SM22_Postcentral_L connectivities alteration after swallowing therapy in brainstem group. FOIS, functional oral intake scale; SM, sensorimotor network.

Brain rs-fMRI showed that in hemispheric stroke patients, changes in the FOIS were related to changes in the functional connectivity of the ventral default mode network of the left precuneus (Table 5 and Figure 2A). However, in brainstem stroke patients, changes in the FOIS were related changes in the functional connectivity of the left sensorimotor network of the left postcentral region. (Table 5 and Figure 2B).

**Table 5:** Correlation between the changes of clinical parameters and altered functional connectivity maps after treatment in hemispheric and brainstem groups.

**Altered functional connectivity maps**	**Changes in 8-point PAS r (*p*-value)**	**Changes in FOIS r (*p*-value)**
**HEMISPHERIC GROUP**		
vDMN (IC15)_ Precuneus_L	0.169 (*p* = 0.619)	0.809 (*p* = 0.003*)
CSMN(IC24)_Cerebelum_Crus1_L	0.376 (*p* = 0.244)	−0.019 (*p* = 0.955)
VSN(IC01)_Parietal_Sup_L	0.268 (*p* = 0.426)	0.327 (*p* = 0.326)
dDMN(IC10)_Fronal_Mid_R	−0.282 (*p* = 0.401)	0.125 (*p* = 0.714)
CSMN(IC24)_Cerebellum_L	−0.559 (*p* = 0.074)	0.154 (*p* = 0.651)
**BRAINSTEM GROUP**		
RSMN(IC09)_Postcentral_R	0.185 (*p* = 0.725)	−0.706 (*p* = 0.117)
vDMN(IC15)_Parietal_Sup_L	−0.185 (*p* = 0.725)	−0.118 (*p* = 0.824)
LSMN (IC22) Postcentral_L	0.339 (*p* = 0.510)	−0.883 (*p* = 0.02*)
vDMN15_Precuneus_L	−0.617 (*p* = 0.192)	−0.235 (*p* = 0.653)
HVN(IC16)_Fusiform_R	−0.432 (*p* = 0.392)	0.294 (*p* = 0.571)
AN(IC21)_Temporal_Mid_L	−0.309 (*p* = 0.552)	−0.794 (*p* = 0.059)
PCCN(IC23)_Parietal_Inf_L	−0.339 (*p* = 0.510)	0.088 (*p* = 0.868)
PVN(IC07)_Cerebellum_L	0.463 (*p* = 0.355)	0.088 (*p* = 0.868)
PVN(IC07)_Calcarine_R	0.772 (*p* = 0.072)	−0.530 (*p* = 0.280)

IC, independent components; vDMN, ventral default mode network; RSMN, right sensorimotor network; CSMN, cerebellar sensorimotor network; VSN, visuospatial network; dDMN, dorsal default mode network; HVN, high visual network; AN, auditory network; PCCN, precuneus network; PVN, primary visual network; LSMN, left sensorimotor network; PAS, penetration-aspiration scale; FOIS, functional oral intake scale. *p < 0.05.

## Discussion

To the best of our knowledge, this represents the first research study to explore the relationship between brain connectivity network changes in rs-fMRI and clinical swallowing functional recovery following swallowing therapy in subacute hemispheric and brainstem stroke patients with oropharyngeal dysphagia. Our findings revealed that swallowing therapy led to a significant improvement in total FDS, FOIS scores in patients with hemispheric and brainstem stroke, and in 8-point PAS score in patients with brainstem stroke. Our research also showed that in patients with hemispheric stroke, the reduced brain connectivity of the ventral default mode network of the left precuneus was related to improvements in FOIS after swallowing therapy. In patients with brainstem stroke, the reduced left sensorimotor connectivity of the left postcentral network was associated with improvements in FOIS after swallowing therapy.

Both hemispheric and brainstem stroke patients with oropharyngeal dysphagia received swallowing therapy and achieved clinical benefits in terms of their swallowing function. In addition, brainstem stroke impaired the movements of the larynx, pharynx, vocal fold, or cricopharyngeal muscles during the pharyngeal phase of swallowing (Martin and Sessle, 1993; Carnaby et al., 2006; Chenji et al., 2016). In the present study, we observed a worse 8-point PAS score (based on VFSS findings) before intervention in patients with brainstem stroke. Therefore, this particular cohort of patients showed significant improvement after training; this was due to the patients developing better muscle coordination during the pharyngeal phase and reducing the severity of their aspiration. These mechanisms also reduced further pulmonary complications.

In earlier studies, associated cortical and sub-cortical regions involving in swallowing had been demonstrated (Teismann et al., 2009, 2011; Luan et al., 2013; Kober et al., 2015) in stroke patients with dysphagia. Li et al. (2009) reported that disrupted functional brain networks related to swallowing motor control in acute stroke patients with dysphagia according to the findings of rs-fMRI study. Some researchers investigated brain imaging using fMRI for acute and chronic stroke patients with dysphagia and they proposed that recovered swallowing were associated with increase of cerebral activation in the contralateral cortex of the intact hemisphere and ipsilateral anterior cerebellum (Li et al., 2009; Mihai et al., 2016). Another research study (Chenji et al., 2016) showed that increased levels of DMN connectivity in patients with greater disability and that this connectivity decreased when performing goal-oriented tasks; consequently, DMN connectivity could be viewed as a baseline functional network. We considered that better swallowing function could lead to reduced levels of DMN connectivity while swallowing. In our present study, we found similar results (Li et al., 2009; Chenji et al., 2016) in that our patients with hemispheric stroke showed improved swallowing function following therapy and that this was associated with reduced brain function network connectivity.

In normal subjects, Babaei et al. (2013) and Malandraki et al. (2009) used brain fMRI to demonstrate increased activation in the cingulate, insula, sensorimotor cortex, prefrontal and parietal cortices, cerebellum, and thalamus during swallowing. Furthermore, Vahdat et al. (2011) observed that alterations in the functional connectivity of the sensorimotor network may be related to motor learning and the importance of the role of somatosensory region in swallowing had been gradually discovered (Babaei et al., 2013; Dehaghani et al., 2016; Mihai et al., 2016). In the present study, stroke patients received a variety of treatments to improve their swallowing, including motor functional training, thermal stimulation, postural compensation, oropharyngeal exercise, compensatory techniques, and swallowing maneuver. We also observed some correlations between clinical swallowing improvements and the SMN network after swallowing therapy in patients suffering from brainstem stroke.

There were several limitations to our study that should be considered when interpreting our conclusions. Firstly, only a small number of patients participated in the study. Secondly, the detailed techniques involved with swallowing interventions were not recorded and compared between hemispheric and brain stem stroke patients. Thirdly, we did not apply longer swallowing interventions for moderate to severe oropharyngeal dysphagia in stroke patients or follow-up the long-term effect of swallowing therapy on swallowing function and neuroplasticity in stroke patients with oropharyngeal dysphagia. In addition, the number of males was 4–5 times as females were enrolled in this study, and the results might be biased by the gender ratio. Finally, we did not recruit stroke patients with oropharyngeal dysphagia in the control group to account for their spontaneous neural recovery, which could have contributed to the alternations in brain fMRI.

## Conclusion

In summary, there was both significant recovery of swallowing function after swallowing therapy for subacute stroke patients with different oropharyngeal dysphagia. The clinical improvements in FOIS were associated with default mode network in hemispheric stroke and with sensorimotor network in brainstem stroke characterized by fMRI, respectively. For these two dysphagic stroke groups with corresponding etiologies, swallowing therapy could contribute to different functional neuroplasticity, which might serve as an indicator for further prognostic evaluation.

## Ethics statement

Institutional Review Board in Chang-Gung Memorial Hospital approved the study protocol, and all of the participants or their guardians provided written informed consent. All of the participants or their guardians provided written informed consent.

## Author contributions

Y-CH and W-CL designed the study, participated in data collection, data analysis and interpretation, writing and revising manuscript and final approval of manuscript. T-WH participated in patient enrollment, data collection, data analysis, and data interpretation. H-CH conducted the data statistical analysis, data interpretation, and manuscript drafting. C-PL participated in patient assessment, data analysis and data interpretation. All authors reviewed the manuscript, contributed to its revision, and approved the final version submitted.

### Conflict of interest statement

The authors declare that the research was conducted in the absence of any commercial or financial relationships that could be construed as a potential conflict of interest.
